# Managing inventories for perishable e-groceries: The value of probabilistic information

**DOI:** 10.1371/journal.pone.0343935

**Published:** 2026-04-09

**Authors:** David Winkelmann, Matthias Ulrich, Michael Römer, Roland Langrock, Hermann Jahnke

**Affiliations:** 1 Department for Empirical Methods, Bielefeld University, Bielefeld, North Rhine-Westphalia, Germany; 2 Department for Management Science and Business Analytics, Bielefeld University, Bielefeld, North Rhine-Westphalia, Germany; Istinye University: Istinye Universitesi, TÜRKIYE

## Abstract

E-grocery retailing allows customers to order products online for delivery within a chosen future time slot. To remain competitive, retailers aim to meet high customer expectations regarding product availability by strategically setting very high service level targets. However, maintaining excess inventory incurs holding costs and leads to spoilage of perishable products, with associated environmental impacts. Retailers face multiple sources of uncertainty, including stochastic customer demand, stochastic spoilage, and potential supply shortages. This renders the determination of optimal replenishment quantities both challenging and crucial for long-term business growth. Fortunately, comprehensive new data sets routinely collected by retailers enable a data-driven approach to controlling inventory levels. This approach includes predictive and prescriptive analytics to (1) estimate suitable underlying probability distributions to represent the inherent uncertainty in the inventory process and to (2) integrate those forecasts into a comprehensive multi-period optimisation framework. In this paper, we propose a stochastic lookahead policy to solve the corresponding optimisation problem, thus supporting the retailers’ inventory management decisions by minimising expected costs while maintaining a specified service level target. By explicitly deriving the value of probabilistic information, we provide guidance for retailers on which sources of uncertainty warrant investments in data collection and processing.

## 1 Introduction

Electronic groceries (e-groceries) encompass click-and-collect services and attended home delivery, i.e., the additional service of delivering products from the retailer to customers [1]. The tight profit margins in grocery retailing also apply to e-groceries. From the supply perspective, e-grocery retailers additionally face high fulfilment costs [2,3]. These costs typically cannot be fully passed on to price-sensitive customers through delivery fees [4]. Despite this challenge, the accelerated growth in e-grocery demand in the wake of the COVID-19 pandemic motivates further investment in already expanded capacities ([5–7]).

This paper is inspired by the business case of a major European e-grocery retailer that offers perishable stock-keeping units (SKUs) with a (stochastic) shelf life spanning multiple demand periods (for details see Supporting Information A in S1 Appendix; [8] describe an e-grocery retailing practice case). Consequently, excess inventory can be sold within a limited number of subsequent demand period(s), affecting replenishment order decisions for those periods [9]. In case of excess inventory, the retailer incurs operational costs for holding or, if the units deteriorate, spoilage. On the other hand, excess demand results in short-term lost sales and potentially long-term customer churn [10]. Although customers benefit from e-grocery retailing by avoiding physical store visits, the requirement to stay home during the delivery process is inconvenient. This increases the likelihood of customers cancelling the whole (virtual) shopping basket in the event of stock-outs. Typically, backordering is also neither possible nor meaningful in grocery retailing, implying that unfulfilled demand is considered lost [11]. Thus, retailers face a trade-off between operational costs associated with excess inventory, driven by holding and spoilage, and customer dissatisfaction resulting from excess demand, which incurs implicit but severe shortage costs. Addressing this trade-off, the e-grocery retailer under consideration operates with very high service level targets of 97% to 99%, aiming to meet long-term strategic objectives, which adds pressure to the low margins in grocery retailing [12].

From a methodological perspective, the retailer faces inter-period dynamics and multiple sources of uncertainty that affect inventory levels, including demand, shelf life, and the quantities delivered by suppliers. This results in a convolution of the probability distributions reflecting these uncertainties. In retail business, the underlying probability distributions are non-stationary but typically depend on exogenous variables (features), such as the weather, and endogenous decisions, e.g., the price for the SKU, which influence demand [12]. In particular, the service level requirements necessitate the application of complex forecasting methods to accurately determine the associated extreme quantiles in the right tail of the demand distribution [12]. The uncertainties mentioned above are typically exacerbated by lead times of multiple days. Therefore, costs associated with a specific order decision are uncertain. To support operational order decision-making, it is essential to effectively capture the business environment through an appropriate inventory model capable of integrating these forecasts. This results in a dynamic stochastic inventory problem – which is notoriously difficult to solve [13]. [14] demonstrate that this particularly holds for perishable inventory systems even when the lead time is fixed and known.

New types of data available in (e-)grocery allow for data-driven approaches in operational processes. [15] underscores the importance of integrating *relevant* information for a successful inventory management process. Specifically, according to [16], the application of advanced analytics can increase grocers’ earnings by 2%. E-grocery retailing provides a fertile ground for demonstrating the impact of comprehensive data. In particular, the availability of uncensored demand data enhances the accuracy of forecasts for future customer demand [12]. Additionally, *advance demand information* (cf. [17]) can potentially reduce fulfilment costs [7] and increase profits [18] for perishable products. While the benefit of data-driven inventory management has already been demonstrated [19], recent approaches mostly focus on modelling uncertainty in customer demand only, while making restrictive assumptions regarding other sources of uncertainty, as within the newsvendor model (see, e.g., [20]). Such models lack the complexity to accurately represent the business case of a retailer dealing with perishable products saleable over multiple but limited periods [21]. Specifically, given the very high service level targets in e-grocery retailing, ignoring potential starting inventories within the newsvendor model amplifies the risk of spoilage with all its negative financial and environmental consequences [22].

This paper aims to explicitly derive the value of probabilistic information for managing inventories in e-grocery retailing. To achieve this, we address two interrelated aspects of operational decision-making stemming from the business environment under consideration: (1) formulating an adequate inventory management model that overcomes limiting assumptions, and (2) integrating probabilistic information into the recurring and time-critical decision-making process. We propose a flexible multi-period inventory management framework that explicitly enables the consideration of perishable SKUs with a stochastic shelf life of multiple periods. This aims to facilitate a data-driven inventory management process that meets the requirements of a real-world e-grocery retailing business case. The model is solved using a Monte Carlo-based approximate dynamic programming approach that determines replenishment order decisions by minimising expected costs over a set of sample trajectories that span a given lookahead horizon. An advantage of this approach, which can be characterised as a *stochastic lookahead policy* following the terminology proposed by [23], lies in the ability to integrate distributional information of stochastic variables available to the decision-maker while, on the other hand, meeting the requirement of limited processing times. The potential of such approximate numerical methods for complex inventory control problems in general is highlighted by [24]. Specifically, [25] demonstrate the successful applicability of a stochastic lookahead policy for the integrated inventory and routing problem faced by an agri-food e-commerce platform.

We evaluate our policy in a simulation-based setting that mimics typical patterns from real-world retailing data; this allows us to assess the benefit of incorporating uncertainty information in isolation, i.e., without additional noise induced by the need to estimate relevant probability distributions. We test our approach against a myopic benchmark from the literature and a deterministic benchmark. Our results allow retailers to compare the savings achieved by integrating probabilistic information with the costs associated with data collection and processing for each source of uncertainty individually. This addresses the question of data prioritisation and value creation, identified as a research gap in previous retailing literature, with the aim of targeted data utilisation [19]. We further discuss the sensitivity of the results with respect to the specification of different model parameters to allow for a generalisation of our findings. Overall, the paper contributes to the interplay of operational decisions, customer outcomes, and retailing costs, identified as an area of opportunity in the empirical analysis of digital retailing research by [6].

## 2 Literature review

This section discusses challenges and opportunities of e-groceries, with a focus on the value of new types of data, before considering its implications for inventory models in grocery retailing.

### 2.1 Challenges and opportunities of e-grocery retailing

In e-grocery, customers order groceries online, and the retailer delivers the purchase directly from local distribution warehouses to the customer. [26] provide an extensive literature review on logistics strategies in e-grocery retailing. In contrast to brick-and-mortar retailing, e-grocers face additional challenges that arise from particular aspects related to warehousing and order picking (see, e.g., [27]) as well as the management of delivery slots (see, e.g., [28]). At the same time, there are specifics regarding the inventory management process that will be discussed in the following.

Due to the additional operational processes (order picking and delivery), there is a longer interval between when a replenishment order for an SKU is placed and when it becomes available to the customer. This reduces the forecasting accuracy of crucial variables, such as the demand distribution for the period under consideration. Specifically, some features used to predict this distribution become less informative the further in advance they are considered. Moreover, the likelihood of customers cancelling the whole (virtual) shopping basket in the event of stock-outs is much higher compared to traditional brick-and-mortar retailing. The paramount convenience of online shopping, namely not having to visit a physical store, may then be outweighed by the inconvenience of needing to place a second order and the general requirement to remain at home during the delivery time slot. In addition, an out-of-stock situation that occurs after the purchase requires an effective substitution policy to mitigate customer dissatisfaction [29]. Consequently, (implicit) costs of excess demand, consisting of both short-term lost revenue and consequences of long-term customer churn, are much higher than operational costs for excess inventory [12]. This asymmetric cost structure, which accounts for customer satisfaction and long-run objectives that influence expected future sales, strongly affects the selection of strategic service levels [30]. Overcoming these challenges in e-grocery retailing necessitates applying complex forecasting methods to accurately determine an extremely right-tail quantile of the demand distribution [12].

At the same time, new types of data provide the opportunity to apply data-driven methods to tackle problems in e-grocery retailing. E-grocers differ in their policy when stock-outs become observable by the customer [31]. In the business case under consideration, customers receive in-stock information only at the final step of the ordering process, yielding uncensored demand data; this allows retailers to monitor customer preferences more accurately [12]. Furthermore, such data enables the explicit quantification of sales actually lost — something not possible with check-out data resulting from traditional brick-and-mortar retailing — leading to a more accurate calculation of costs associated with a specific replenishment order quantity. Additionally, customers can select a delivery slot up to fourteen days in advance. This provides information on *known demand*, the amount of customer demand for a future delivery period that is already known to the retailer when a replenishment order quantity must be determined. For example, [32] make use of such data by proposing a classification-based model selection for demand forecasting in e-grocery retailing. Previous literature also considers the case of *advance demand information*, suggesting that customers may exhibit a higher willingness to pay for shorter delivery lead times [17]. Translating these findings to grocery retailing, [7] analyse how demand information can reduce fulfilment costs. [18] build a subscription-based model and demonstrate that retailers can increase revenue by offering price discounts to customers willing to provide advance demand information for perishable products. By reducing uncertainty in customer demand, such data also has the potential to mitigate spoilage while maintaining a specific service level target.

Given the abundant data available in retailing, [19] discuss how retailers can focus on specific data with the highest potential to impact business. In the business case under consideration in this paper, the retailer has to deal with various sources of uncertainty in the inventory process. Therefore, an effective and targeted utilisation of data requires determining which variables warrant investments in collecting and processing distributional information. In the field of decision analysis, the enhancement in expected performance achieved through the use of distributional information is referred to as *expected value of including uncertainty* (EVIU). For a detailed description of EVIU, and its relation to the value of information in economics, see, e.g., [33]; a study addressing the value of information in the context of grocery retailing has been performed by [7]. In the field of stochastic programming, the concept of EVIU is commonly known as *value of the stochastic solution* (VSS), see, e.g., [34]. While most studies examining EVIU and VSS compare the consideration of distributions for *all* stochastic variables to using no distributions at all, our investigation focuses on evaluating the value of including distributions for each subset of stochastic variables. These results can be weighted against the costs associated with collecting and processing the data required in the business case to obtain distributional information for the respective stochastic variable(s). In particular, this enables the retailer to decide whether adopting a probabilistic representation is worthwhile for each source of uncertainty.

### 2.2 Inventory models for grocery retailing

The scientific study of inventory control problems has a long tradition that dates back to the EOQ model by [35], with its restrictive assumptions of fixed and deterministic demand as well as unlimited shelf life. Since then, extensive research has been conducted to determine optimal replenishment order quantities for different settings. To accurately represent the business environment under consideration in this paper, we need to formulate a periodic review inventory model that, firstly, considers lost sales and non-zero lead time and, secondly, applies to perishable SKUs.

For the backorder case, i.e., if demand can be fulfilled in subsequent periods, an order-up-to policy is known to be optimal for non-perishable SKUs [36]. However, due to complexity considerations, there is limited knowledge about optimal replenishment policies for the more realistic case of periodic-review lost sales models, even for non-perishables (refer to the review on inventory models with lost sales by [13]). In a more recent review, [24] state that inventory models with lost sales generally cannot be solved using exact methods due to the associated extensive size of the state space. Although in specific cases and under restrictive conditions the optimal order quantity in the lost sales case can be approximated by backorder models [37], cost deviations can be as high as 30% [38].

Most SKUs in grocery retailing have a finite shelf life spanning multiple periods, leading to operational costs either for inventory holding (if units can be carried over to the following period) or spoilage and stock reductions (if they are not sold before their shelf life expires; see, e.g., [7]). These reductions in the inventory level must be considered when making replenishment order decisions. [39] provide an overview of trends in managing inventories for such perishable products; more recently, [40] explicitly discuss discrete review multi-period inventory models for this case. Most literature addressing finite shelf lives assumes that the number of sales periods is fixed and known (e.g., [41–43]). The same assumption is made by [14], who develop a periodic-review lost sales model for perishable products with non-stationary demand, relaxing the assumption of independent and identically distributed demand. [44] extend this approach to positive lead times. Both papers highlight that such models are notoriously difficult to solve due to the curse of dimensionality and thus propose approximate inventory control policies. While [45] consider the case of SKUs that decay at a constant rate, for perishable products like fruits and vegetables, it is more realistic to treat the number of sales periods as a random variable. The associated probability distribution can be estimated by modelling the decay of the SKUs over time. This decay can be described by a constant fraction of the given inventory or by a rate that changes according to an underlying function [46], such as the cumulative distribution function of an exponential distribution [47].

Regarding the demand process, some of the literature relies on relatively simple modelling approaches, such as employing a Poisson process for the arrival of customer demand (see, e.g., [7]). While such restrictive assumptions facilitate the derivation of optimal replenishment order policies, they often lack the flexibility to capture typical non-stationary demand patterns observed in real-world retailing data. In fact, [48] had already proposed using a negative binomial distribution for modelling customer demand in retailing. Concerning the non-stationary nature of the demand process, [32] highlight the importance of a case-specific estimation of the demand distribution based on data from an e-grocery business case. Specifically, the combination of high service level targets, aimed at achieving customer satisfaction, and complex demand patterns regularly observed in e-grocery retailing necessitates the use of probability distributions that can account for characteristics such as overdispersion or skewness.

Estimating more complex probability distributions suitable to quantify random demand and simultaneously integrating them into the decision-making process remains crucial in inventory management. Many data-driven approaches that leverage the availability of comprehensive data at low costs (see, e.g., [49–51]; [20]) rely on the simplifying assumptions of the newsvendor model, the classical model for stochastic customer demand [52,53], which assumes a single period and order decision. However, more complex models are required to address the challenges encountered in retail practice [21]. In this regard, [54] propose several extensions to the newsvendor model (see, e.g., [9] for the analysis of a multi-period version).

In most practical settings, retailers additionally face the risk of supply shortages, which may arise from constraints in the distribution channels. This issue is referred to as *random yield* in the literature. Existing literature on supply uncertainty assumes that retailers know their suppliers’ true supply distributions (see, e.g., [55–57]). [58] were among the first to address problems where both supply and demand are random, deriving the optimal order quantity for the unconstrained newsvendor problem with random yield. [59] incorporate non-stationary supply by assuming it follows a Bernoulli process, where supply can either be completely absent or fully available.

Following the literature discussed above, it appears that attempting to solve periodic review inventory control models with multi-period lead times and lost sales for perishable SKUs to optimality is not feasible for real-world problems. Moreover, the business environment considered here requires the integration of stochastic and non-stationary customer demand, shelf lives, and random yield. Consequently, the retailer faces a complex convolution of probability distributions that govern the development of inventory levels during the lead time, rendering the challenge even more complicated [60]. However, [24] emphasise the potential of approximate numerical methods, such as deep reinforcement learning, for addressing complex sequential decision problems like the one at hand. We concur with their assessment and propose an approximate dynamic programming approach for e-grocery inventory management that incorporates the multiple sources of uncertainty outlined above. Several publications address the determination of replenishment quantities for perishable SKUs – such as the work by [22], which presents a periodic-review inventory control model with non-stationary demand and deterministic shelf life. However, to the best of our knowledge, our study is the first to consider a multi-period model for perishable SKUs with lead time accounting for the complete uncertainty resulting from stochastic customer demand, stochastic shelf lives, and random yield.

## 3 Modelling framework and solution approach

A central goal of this paper is to evaluate the value of probabilistic information for the various sources of uncertainty inherent in the inventory management process of an e-grocery retailer. In the following, we introduce a framework for modelling the retailer’s operational decision-making problem and propose a policy for determining replenishment order quantities based on the available data. The framework is based on the events that occur throughout the day, specifically the decision on a replenishment order quantity, the realisation of supply and customer demand, and spoilage at the end of the day. Furthermore, we explicitly outline how we model the stochastic variables — namely demand, spoilage, and supply shortages — and integrate them into our optimisation framework. Finally, we formulate the problem as a sequential decision process and introduce a stochastic lookahead policy that leverages the representation of uncertain parameters as probability distributions to determine replenishment order quantities.

### 3.1 Assumptions and cost structure

The assortment of the retailer under consideration includes several thousand SKUs from various categories, such as fruits, vegetables, and meat, most of which have a very limited shelf life, ranging from one to multiple days, as is typical for grocers [40]. Our modelling approach takes the perspective of a single fulfilment centre, where we assume unlimited storage capacity. In practice, the retailer needs to effectively manage the inventory process daily for various SKUs and several fulfilment centres. Since the retailer controls the stowing and picking processes, we suppose units are picked according to the *First In – First Out* (FIFO) principle, meaning that the oldest SKUs are sold first.

The retailer’s objective is to control inventory levels such that total (expected) costs are minimised. These costs are related to excess inventory, incurring holding costs *v* per unit, spoilage costs *h* per unit, and costs associated with each unit of lost sales *b*. Although it is generally straightforward to specify cost parameters for inventory holding and spoilage, this is much more difficult for lost sales [61,62]. Thus, many retailers operate with a strategic service level target, that is, the percentage of stochastic customer demand that should be fulfilled. Mathematically, under strong model assumptions, each service level corresponds to a fixed ratio between costs for excess inventory and shortage. This allows retailers to implicitly calculate costs associated with one unit of lost sales from the service level strategically determined and known costs for excess inventory ([12]). We derive cost parameters for lost sales based on these considerations, addressing the trade-off between shortage costs and costs incurred by excess inventory (cf. [22]).

### 3.2 Dynamics of the inventory process and resulting cost

**Fig 1:**
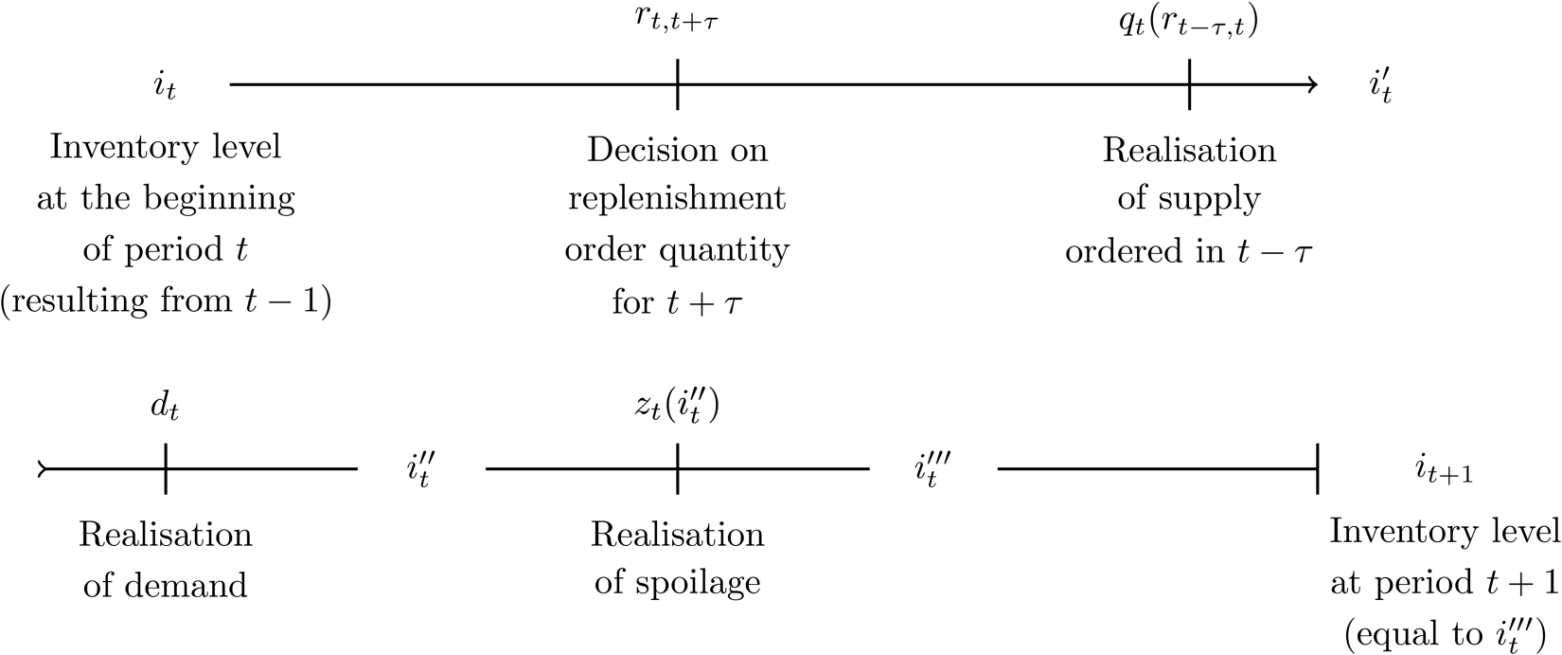
Sequence of events within one demand period.

The inventory *i*_*t*_ of an SKU available at the beginning of the demand period *t* generally comprises units delivered at different replenishment instances. Let ${\tilde{i}}_{t,j}$ denote the number of units available in period *t* that were supplied *j* periods ago. Then, $i_{t}=\sum_{j=0}^{J}{\tilde{i}}_{t,j}$, where *J* corresponds to *t*he maximum shelf life of the SKU. Therefore, the total inventory *i*_*t*_ can be expressed as a vector with elements ${\tilde{i}}_{t,j}$ ([7] use a similar approach). We denote the quantity supplied at the beginning of period *t* as *q*_*t*_ and demand as *d*_*t*_. We assume that the replenishment order quantity $r_{t-\tau ,t}$ to be delivered after a lead time τ canno*t* be adjusted by the retailer after its specification in t−τ. For simplicity, we assume that the intra-period dynamics can be represented by a sequence of events that occur within period *t*, affecting the size of the inventory *i*_*t*_. Primes indicate the changes resulting from these events (refer to the timeline visualisation in Fig 1 and the numerical example in Supporting Information B). At the beginning of period *t*, with starting inven*t*ory *i*_*t*_, the retailer must first decide on the replenishment order quantity $r_{t,t+\tau }$. This is the quantity that affects supply in the future period t+τ. However, the decision needs to be *t*aken without having information about realisations of the stochastic variables in period *t*. Subsequently, supply $q_{t}(r_{t-\tau ,t})$ becomes known, affecting the inventory in the following manner: $i_{t}^{\prime }=i_{t}+q_{t}$. Given that we assume supply to be stochastic, this quantity is uncertain and can deviate from the replenishment decision $r_{t-\tau ,t}$ (for details, see 3.3). In our case, SKUs are picked from the inventory according to a FIFO principle. After taking the (satisfiable) demand *d*_*t*_ out, the new inventory is denoted as $i_{t}^{\prime \prime }$. This forms the basis for *t*he amount affected by deterioration during period *t*; we denote the corresponding realisation of spoilage as *z*_*t*_, leading to the new inventory $i_{t}^{\prime \prime \prime }=i_{t}^{\prime \prime }-z_{t}$. This represents the inventory at the end of period *t* and gives the inventory at the beginning of the following period: $i_{t+1}=i_{t}^{\prime \prime \prime }$.

In the case of lost sales, we obtain the inter-period dependencies as follows:


$$i_{t+1}=\max(i_{t}+q_{t}-d_{t}-z_{t},0).$$ (1)

At the end of each period *t*, costs arise depending on demand *d*_*t*_ and the number of available units of the SKU $i_{t}+q_{t}$. By assuming periodic replenishments, we can disregard fixed order costs in our model. Given the cost structure outlined above, costs incurred during period *t* can be expressed as follows with $\left(x{)}^{+}=\max(x,0\right)$:


$$C(r_{t-\tau ,t})=v\cdot {\left(i_{t}+q_{t}-d_{t}-z_{t}\right)}^{+}+b\cdot {\left(d_{t}-i_{t}-q_{t}\right)}^{+}+h\cdot z_{t}.$$

### 3.3 Modelling uncertainty in the inventory process

Retailers face uncertainty regarding the number of units requested by customers in period *t*, rendering demand a random variable *D*_*t*_ with cumulative distribution function (CDF) $F_{D_{t}}$. Additionally, taking into account potential supply shortages, the quantity delivered by the supplier, *Q*_*t*_, becomes stochastic; specifically, it depends on the quantity ordered $r_{t-\tau ,t}$. While we assume that the number of units delivered cannot exceed the order quantity, the retailer may experience a shortfall in the actual delivery quantity ($q_{t}\leq r_{t-\tau ,t}$). If the relative supply shortage were known and constant, a retailer could easily adjust the specified replenishment order quantity by adding the known percentage of shortage to derive the target order quantity. However, in retail practice, supply shortages are neither constant nor known; instead, they follow an unknown probability distribution. In our model, we consider three different supply states *G*_*t*_: complete delivery (state 1), a cancellation of the total delivery (state 2), and partial delivery (state 3). These states determine the relative proportion $\delta_{t}$ of ordered supply that was realised in each demand period *t*. Since supply shortages often result from persistent issues within the supply chain, we model the sequence of supply states using a homogeneous Markov chain characterised by transition probabilities and its s*t*ationary distribution. In the case of partial delivery, we assume that the proportion of units supplied follows a beta distribution, with additional point masses at zero and one, respectively ([63]). For technical details, see Supporting Information C in S1 Appendix.

To capture the case of uncertain shelf lives, we model the shelf life of an SKU in days using an empirical discrete distribution. As elaborated in detail in Supporting Information D, this empirical distribution allows us to derive the conditional probability of a unit of the SKU deteriorating after a certain number of days, given that it was still saleable in the preceding period. We denote the (stochastic) total number of deteriorated units at the end of period *t* by *Z*_*t*_. Note that in case of a positive fixed lead time τ>0, the inventory *I*_*t*_ at the beginning of period *t* is unknown at the decision instance t−τ when *t*he order $r_{t-\tau ,t}$ must be placed. Instead, *I*_*t*_ depends on demand $D_{t-\tau },D_{t-\tau +1},\ldots ,D_{t-1}$ as well as the random yield and spoilage that occur during the lead time. Consequently, the distribution of inventory *I*_*t*_ is a convolution of the corresponding probability distributions.

Considering all stochastic variables that affect the inventory level, we can express the expected costs for period *t* in terms of the specified distributions as follows:


$$E\left[C(r_{t-\tau ,t})\right]=v\cdot E{\left[I_{t}+Q_{t}-D_{t}-Z_{t}\right]}^{+}+b\cdot E{\left[D_{t}-I_{t}-Q_{t}\right]}^{+}+h\cdot E\left[Z_{t}\right].$$ (2)

Due to the lead time τ, the replenishment order decision $r_{t-\tau ,t}$ taken in period t−τ does not affect the cost in period t−τ but only influences the cost accumulated starting in period *t*. In this work, we aim to simultaneously consider consecutive periods affected by the replenishment order decision [64]. Given the system dynamics in Eq (1), minimising the total expected costs over a planning horizon *T*,


$$\min_{r_{1,1+\tau },\ldots ,r_{T-\tau ,T}}\sum_{t=1}^{T-\tau }E[C(r_{t,t+\tau })],$$ (SDLI)

yields the periodic review stochastic dynamic lost-sales inventory model with lead time (SDLI) under consideration in this paper.

### 3.4 Formulating the problem as a sequential decision process

Besides proposing a solution procedure for deriving a replenishment policy for the inventory model (SDLI), supporting replenishment order decisions in e-grocery retailing particularly requires appropriately responding to relevant data that becomes available in the business environment. In our case, this data includes daily realisations of the inventory level, supply, customer demand, and spoilage. Consequently, it is necessary to rerun the solution procedure for the inventory model (SDLI) every day and for each of the various SKUs based on newly available information. This proceeding transforms (SDLI) into a decision model that is embedded within a sequential decision process, based on the model elements described in Sections 3.2 and 3.3; here, we adopt the terminology and notation conventions proposed by [65] and adapted by [66].

The core elements of a sequential decision process operating at the level of demand periods, based on the data becoming available over time, are an information model Ω, encompassing exogenous information that is stochastic for future periods, and a decision model, in our case (SDLI). Realisations of the information model at the end of period *t* (demand, spoilage, and supply) are denoted by $\omega_{t}$ and determine the resul*t*ing costs for this period. In retail practice, parts of these realisations, along with contextual information, are fed into prediction models that provide estimates for the parameters of the underlying (non-stationary) probability distributions of the stochastic variables within the decision model. Indeed, the stochastic and dynamic decision model (SDLI) introduced here necessitates the incorporation of estimated probability distributions within $\omega_{t}$. Given the availability of comprehensive data sets in the e-grocery business environment, this poses both an opportunity and a challenge for descriptive and predictive analytics [67].

The decision model utilises a state *s*_*t*_ and yields a decision *x*_*t*_. In this context, the state *s*_*t*_ encompasses several components: the inventory *i*_*t*_ (along with the corresponding vector ${\tilde{i}}_{t}$ indicating the supply dates, as introduced in Section 3.2), the supply state from the previous period $G_{t-1}$, the set of ordered (but not yet delivered) replenishment quantities $r_{t-\tau ,t},\ldots ,r_{t-1,t+\tau -1}$, and the (estimated) probability distributions of customer demand, spoilage, and yield of future periods. Solving the decision model results in a post-decision state $s_{t}^{x}$, incorporating the newly determined replenishment order quantity $r_{t,t+\tau }$, and determines expected costs for the period under consideration. Given the state *s*_*t*_, a decision *x*_*t*_, and the realisation of the information model $\omega_{t}$, a transition function *T* defines the state for the following period: $s_{t+1}=T(s_{t},x_{t},\omega_{t})$. The state variable *s*_*t*+1_ also includes the current supply state *G*_*t*_, the set of replenishment order quantities augmented by the newly determined $r_{t,t+\tau }$, and the estimated probability distributions of the various random variables that will affect the future inventory level.

**Fig 2:**
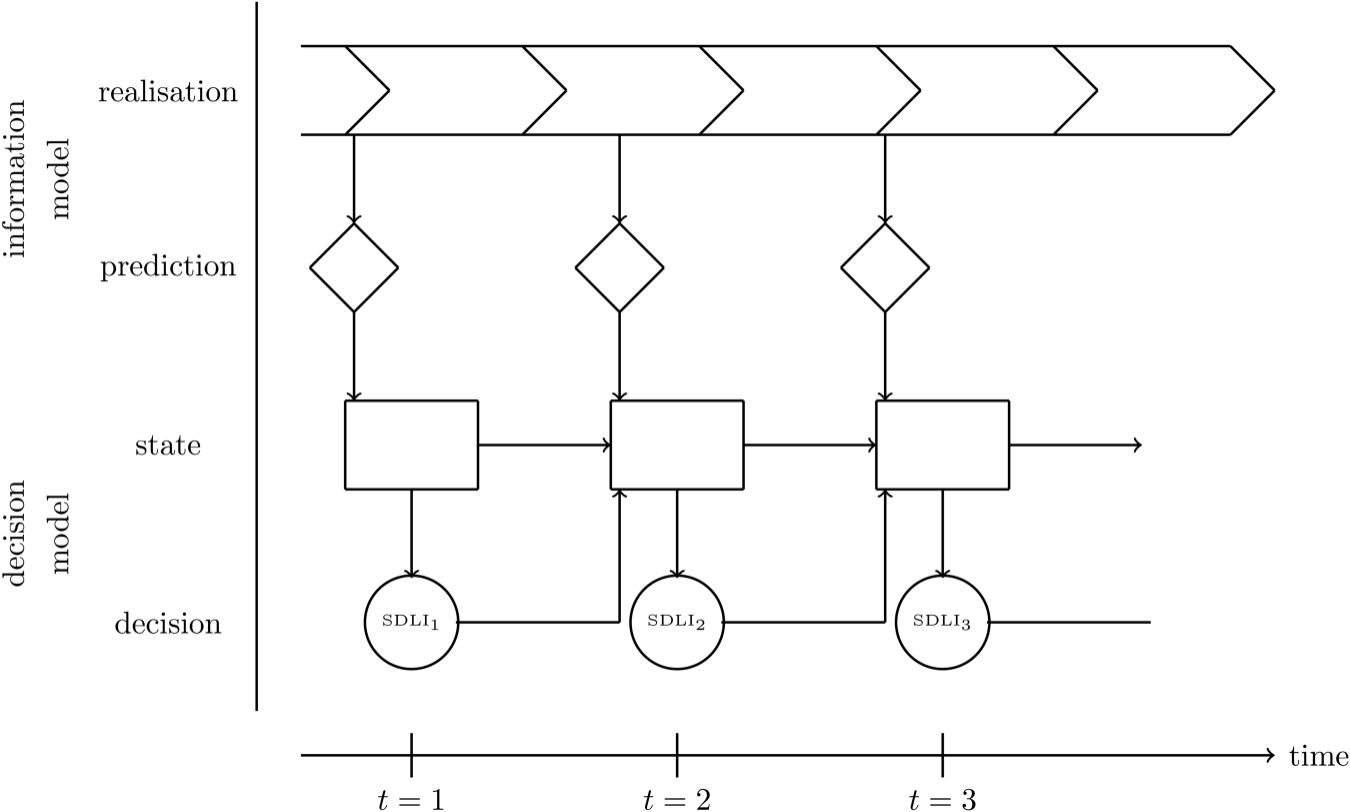
Representation of our sequential decision process adapted from [68].

The decision *x*_*t*_, in our context determining a replenishment order quantity $r_{t,t+\tau }$, is induced by a policy π: $x_{t}=X^{\pi }(s_{t})$. The goal is to identify an optimal policy $\pi^{*}$, that is, a predefined reaction aimed at minimising the (expected) costs resulting from a decision $r_{t,t+\tau }$ over a specified planning horizon. These costs comprise the expected immediate costs, i.e., costs for period t+τ where the decision affects the inventory management process, and the expected sum of future costs depending on the post-decision state $s_{t}^{r}$, denoted as the *value of the post-decision state* $V(s_{t}^{r})$. Given the significance of data-driven estimation of the relevant probability distributions in the sequential decision process within the e-grocery sector, we adapt the representation proposed by [66] accordingly (see Fig 2).

For a given planning horizon *T* and a policy π, we can express the sum of expected immediate and future costs to be minimised in period *t* as follows:


$$E[C_{t,t+\tau }^{\pi }]=E[C(s_{t},r_{t,t+\tau }^{\pi })]+\underset{V(s_{t}^{r})}{\underbrace{E[\sum_{j=t+1}^{T-\tau }C(s_{j},r_{j,j+\tau }^{\pi }(s_{j})|s_{t},r_{t,t+\tau }^{\pi })]}}.$$ (3)

Here, the expected immediate costs incurred by a policy π that leads to the decision in period t−τ can then be written using the estimated probability distributions of the uncertain quantities (as detailed in Eq (2)); note that these estimates depend on the state $s_{t-\tau }$.

### 3.5 A lookahead-based decision policy

Solving the decision models necessitates an order policy π that minimises the expected cost for a given state *s*_*t*_ (see Eq (3)). According to [24], the most effective approach for addressing complex inventory control problems under uncertainty is to utilise approximate numerical methods. Specifically, their discussion highlights the use of deep reinforcement learning (DRL) techniques, which employ deep neural networks (DNNs) to approximate either the value of taking a particular decision in a given state or an optimal policy function. Following the policy classification proposed by [65], this approach can be categorised as a value function approximation (VFA)-based policy or as a policy that relies on policy function approximation (PFA). However, in our context, we opted against relying on learning a VFA or a PFA, as this would entail training more than 100 ML models (e.g. DNNs) for a business case. Moreover, [24] demonstrate that DRL approaches tend to perform very well for stationary problems; e-grocery inventory management typically operates in highly non-stationary environments. The non-stationarity is only partly explainable by regular effects such as seasonality. Consequently, applying a DRL-based policy would require frequent and costly re-training of the VFA/PFA models, making it less practical for our specific application.

Instead of employing a learned VFA model, we propose approximating the value of an order decision using a Monte Carlo simulation with a limited lookahead horizon *H*. Following [65], the resulting policy can be characterised as a stochastic lookahead policy. A key advantage of this type of policy is that, according to the terminology introduced by [66], it internally utilises the information model. This allows the policy to adapt naturally to (even structural) changes in the information model without retraining an approximation model. Additionally, since the policy is based on sampling, it can be seamlessly combined with advanced and context-dependent distributional forecasting methods, such as those proposed by [32].

Given the lookahead horizon *H*, the order decision taken at *t* affects the objective function only in period t+τ, i.e., when the order is supposed to be delivered. Therefore, we set H≥τ and denote the number of lookahead periods exceeding τ by ν, that is, H=τ+ν. In case of H=τ (that is, ν=0), we can approximate the expected cost $E\left[C(s_{t},r_{t,t+\tau }^{\pi })\right]$ in period t+τ for a given state *s*_*t*_ and replenishment order decision, induced by the policy π, $r_{t,t+\tau }^{\pi }$, by averaging over *N* simulated sample paths starting at period *t* and ending at period t+τ. For a sample path *n*, the cost $C^{n}(s_{t},r_{t,t+\tau }^{\pi })$ is obtained by simulating the state-transition logic outlined in Section 3.2. This simulation begins with the initial state *s*_*t*_. It utilises the given decision $r_{t,t+\tau }^{\pi }$, along with random samples drawn from the distributions that represent supply, demand, and spoilage in each simulated period from *t* to t+τ. In this context, the optimisation problem to be solved in each period *t* can be formulated as follows:


$$E(C(s_{t},r_{t,t+\tau }^{\pi }))\approx \frac{1}{N}\sum_{n=1}^{N}C^{n}\left(s_{t},r_{t,t+\tau }^{\pi }\right).$$

If our lookahead horizon H>τ, that is if ν>0, we extend the sample paths described above until the final period *t* + *H* of the lookahead horizon. This extension enables us to capture the effects of the order decision on demand periods beyond t+τ more accura*t*ely. The costs incurred in the lookahead periods following t+τ are affected not only by the decision $r_{t,t+\tau }$ to be taken in *t* but also by the ’simulated’ decisions $r_{j,j+\tau }$ taken in periods *j* with t≤j≤t+ν that are part of the lookahead. To account for the diminished precision of forecasts for future periods and to reflect the relative decrease in the importance of *t*he decision $r_{t,t+\tau }$, we weight the expected costs by the factor $\rho^{j-(t+\tau )}$, where ρ∈(0,1) for periods j≥t+τ. Note that while the lookahead decisions $r_{j,j+\tau }$ for *j* > *t* are not implemented, they still contribute to the objective function of the decision model (SDLI). Thus, the objective used to determine the lookahead policy reads as follows:


$$\min_{r_{t,t+\tau },\ldots ,r_{t+\nu ,t+\tau +\nu }}\left(\frac{1}{N}\sum_{n=1}^{N}(C^{n}(s_{t},r_{t,t+\tau }^{\pi })+\sum_{j=t+1}^{t+\nu }\rho^{j-t}\cdot C^{n}(s_{j}^{n},r_{j,j+\tau }^{\pi }(s_{j}^{n})|s_{t},r_{t,t+\tau }^{\pi }))\right)$$ (4)

The objective does not include costs incurred in periods before t+τ, as these costs are not affected by the decisions involved in the lookahead. To determine the replenishment order quantity $r_{t,t+\tau }$ to be delivered in period t+τ, we aim to find the quantity that minimises average costs over the sample paths, as outlined in Eq (4). To achieve this, we employ a widely established heuristic Nelder-Mead-based numerical optimisation approach. For t<τ, replenishment order quantities $r_{t-\tau ,t}$ are assumed to be given; resulting costs for these periods are not part of the optimisation problem.

## 4 Evaluation of the lookahead policy and the value of probabilistic information

The decision policy introduced above is capable of explicitly integrating uncertainty in the inventory management process, arising from stochastic and non-stationary demand, spoilage, and supply shortages, when determining replenishment order quantities. To provide guidance for retail practice, we consider an example SKU from a dataset provided by a major European e-grocery retailer. However, since the accuracy of the estimates for the probability distributions is highly dependent on the quality of the available data, we use a simulation-based setting to evaluate the proposed lookahead policy. Here, we define the underlying distributions in accordance with our business case as detailed in Supporting Information A. This approach helps us to avoid potential inaccuracies and to facilitate a comprehensive comparison between different policies, allowing us to analyse the importance of incorporating probabilistic information in isolation. In doing so, we consider the simplified situation in which the retailer is aware of the probability distribution for each source of uncertainty.

Previous literature on e-grocery retailing suggests the newsvendor model, with its restrictive assumptions, for determining replenishment order quantities in case of stochastic customer demand (see the literature review in Section 2.2). As a benchmark, we adopt this myopic approach, thereby ignoring the possibility of transferring units to subsequent periods and potential supply shortages. In the second step, we analytically calculate replenishment order quantities in the multi-period setting by considering a deterministic scenario using expected values of the relevant random variables as parameters in the decision model. Finally, we replace these expected values with probabilistic information (represented by distributions) to demonstrate the potential value of the data available on the stochastic variables in the inventory process and to illustrate the advantage of our solution approach. The comparison with a myopic and a deterministic approach follows the recommendation by [69].

### 4.1 Simulation setup: distributions, parameters, and data

We generate an experimental data set that encompasses *T* consecutive demand and supply periods for an example SKU. This data includes information on demand, spoilage, and supply shortages. Considering perishable SKUs with a shelf life ranging from one to multiple periods, we employ a specific parameter vector for the data-generating process in subsequent analyses. Distributions and parameters in the data-generating processes are chosen to resemble the information structure found in the data provided by the business partner (see Supporting Information A in S1 Appendix).

Based on findings from the literature (see, e.g., [48], and for this data in particular, cf. [32]), we assume that the realisations of (uncensored) demand in period *t* follow a negative binomial distribution with mean $\mu_{t}$ and size parameter *k*_*t*_, i.e.,


$$d_{t}^{real}\sim \text{NegBinom}(\mu_{t},k_{t}).$$

**Fig 3:**
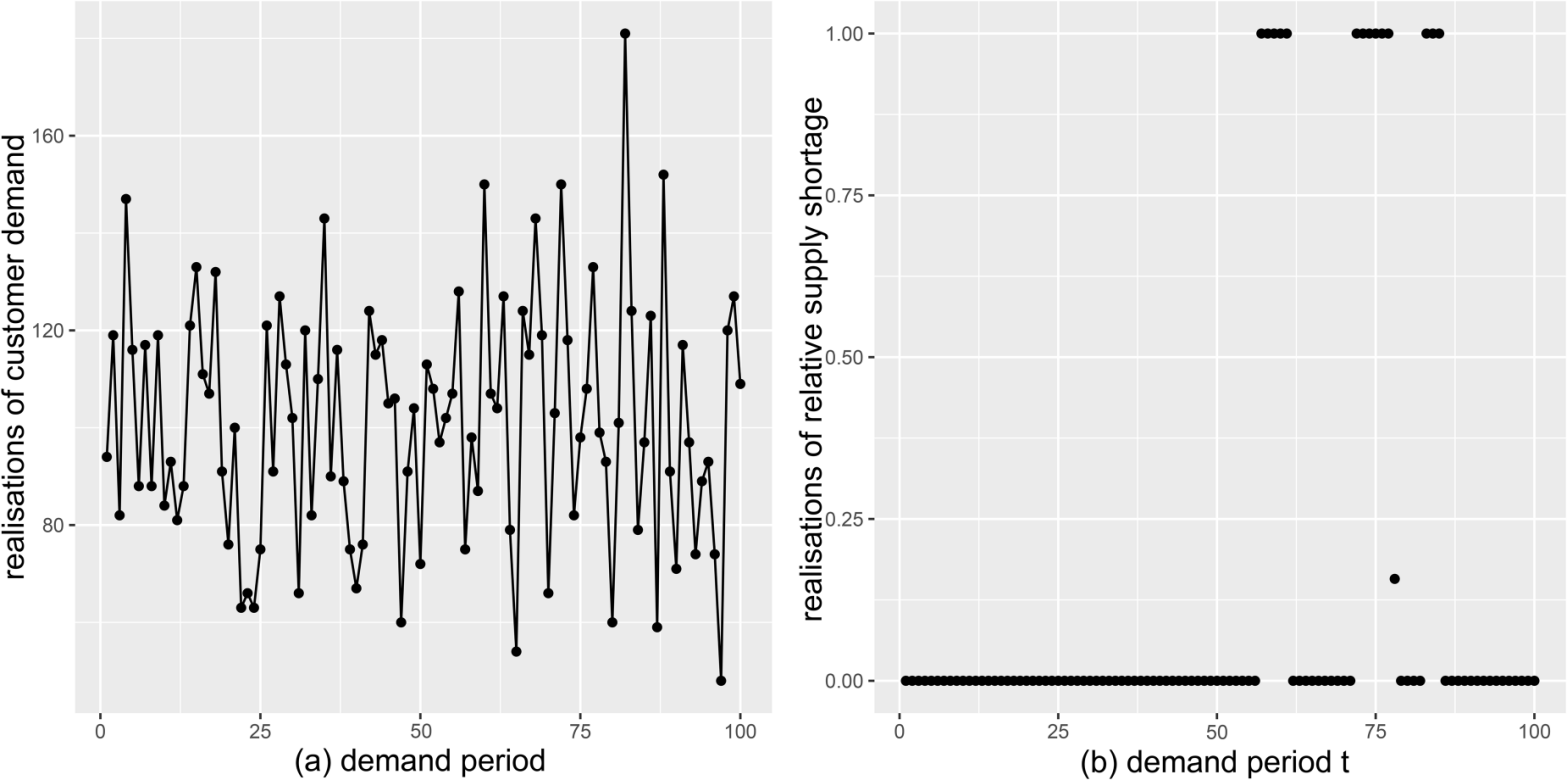
Realisations of demand and shortage for demand period t=1,…,100.

We reparameterise the distribution in terms of its mean $\mu_{t}$ and variance $\sigma_{t}^{2}=\mu_{t}+\kappa_{t}$, where $k_{t}=\mu_{t}/(\sigma_{t}^{2}-\mu_{t})$. To accommodate non-stationary demand, we draw the parameters of the demand distribution for each period as $\mu_{t}\sim Pois(\lambda_{\mu })$ and $\kappa_{t}\sim Pois(\lambda_{\kappa })$. For the subsequent analyses, we assume $\lambda_{\mu }=100$ and $\lambda_{\kappa }=300$. While our model is capable of naturally adapting to structural changes in the information model, such as seasonality, with the chosen probability distributions, we simplify by avoiding such more complex structures, but consider demand to be independent across different periods. In practice, this information can be drawn from probabilistic forecasts, such as those suggested in [32]. An example realisation of simulated demand over 100 periods is shown in Fig 3(a).

The shelf life of the SKU is generated from a distribution with probability mass function *f^sl^*(*j*), as detailed in Table 1. Here, *j* = 1 corresponds to the situation where the unit deteriorates at the end of the delivery period (i.e., day 0). The mean shelf life implied by this distribution is three periods. The conditional probabilities of a unit deteriorating after exactly *j* periods, given it was still saleable at the beginning of that period, are provided in Table D1 in Supporting Information D.

**Table 1:** Distribution of the shelf life in the simulated data set.

*j*	1	2	3	4	5	6
*f*^sl^(*j*)	0.05	0.10	0.15	0.35	0.20	0.15

The sequence of delivery states – namely, complete delivery (state 1), complete shortage (state 2), and partial delivery (state 3) – across demand periods is governed by a Markov chain with the transition probability matrix (TPM)


$$\begin{array}{c} \Theta =(\begin{array}{ccc} 0.99 & 0.005 & 0.005\\ 0.5 & 0.4 & 0.1\\ 0.5 & 0.1 & 0.4 \end{array}). \end{array}$$

The associated stationary distribution, which is also used as the distribution for period *t* = 1, is given by the transpose of $\theta^{*}\approx (0.98,0.01,0.01)$. If the retailer encounters a partial supply shortage, that is, if state 3 is active, the realised relative amount of supply follows a beta distribution with shape parameters α=2 and β=3. This results in an average relative shortage of 60% in case of partial delivery and an overall average shortage of $\bar{\theta }=1.57\%$. An example realisation of relative shortage for demand periods t=1,…,100 is illustrated in Fig 3(b).

In accordance with the strategic environment of e-grocery retailers (see Section 3.1), we assume the cost for one unit of excess demand to be *b* = 5, inventory costs to be *v* = 0.1 per unit and time period, and per-unit spoilage costs to be *h* = 1 for the SKU considered. This relation between excess inventory costs and shortages takes into account the high service-level target in e-grocery retailing. For the lookahead policy, the absolute values of the cost parameters are not relevant; rather, it is the relation between these parameters that influences the solution determined by the model. A lead time of τ=3 days is assumed between the replenishment order decision and the delivery to the retailer’s fulfilment centre. Our evaluation is based on *T* = 5000 simulated periods, which corresponds to more than 15 years of data in a business case. This large number of simulated data points is used to minimise the impact of noise in the comparison. All optimisation experiments are conducted in Python, using the *minimise* function from the *scipy.optimize* package, employing the *Nelder-Mead* method with a tolerance of 10^-9^. The program code to reproduce all simulation results can be obtained from Zenodo [70].

### 4.2 Myopic benchmark: Newsvendor model

In the newsvendor model, it is assumed that each unit of an SKU can be sold for only one demand period, allowing for the optimisation of replenishment order quantities for each period individually. One unit of excess inventory incurs spoilage costs *h*, while each unit of lost sales leads to costs of *b*. The optimal order quantity can be obtained at the *b*/(*b* + *h*)–quantile of the (estimated) CDF of demand *F*_*D*_ [52,53], with $F_{D_{t+\tau }}$ representing the demand forecast made in period *t* for period t+τ. Under these myopic assumptions, the resul*t*ing replenishment order quantity $r_{t,t+\tau }^{*}$ can then be calculated as:


$$r_{t,t+\tau }^{*}=F_{D_{t+\tau }}^{-1}\left(\frac{b}{b+h}\right).$$ (5)

For each period t∈T, we determine replenishment order quantities according to Eq (5). Thus, we assume that the decision-maker disregards the potential transfer of excess units at the end of a demand period, leading to an underestimation of the starting inventory at the beginning of most demand periods, i.e., if there are units transferred. Additionally, the risk of supply shortages is not taken into account when determining replenishment order quantities. At the end of each period, the realised holding of inventory, spoilage, and lost sales generate costs based on the given cost parameters (*h*, *v*, *b*). We then calculate average costs over the considered time horizon *T*.

We obtain an average order quantity of 119.03, an average inventory level of 199.42, and an average amount of spoilage of 17.52, resulting in an average per-period cost of 38.84. The retailer is able to satisfy 99.72% of customer demand, which exceeds the strategic service level target. While this increases customer satisfaction, it leads to higher costs compared to a scenario where the intended service level is precisely met, due to the additional inventory holding and spoilage costs. The deviation from the desired service level is attributed to the newsvendor models’ neglect of inter-period dependencies when determining replenishment order quantities. To balance high product availability and costs for excess inventory, more sophisticated models that account for the various stochastic factors in e-grocery retailing are necessary.

### 4.3 Deterministic benchmark: Expected values

We now account for the dynamic nature of the inventory management problem by considering that SKUs have an expected shelf life spanning multiple periods. Specifically, the expected shelf life is three periods, with variation as detailed in Table 1. In addition, we consider the risk of supply shortages. Due to these two additional sources of uncertainty, the newsvendor model is no longer applicable. To still derive solutions analytically, we rely on deterministic expected values for the stochastic variables that affect the inventory management process, namely demand, spoilage, and supply shortages. Consequently, we continue to ignore the stochastic variation in supply shortages and shelf life, and, compared to the newsvendor approach, also disregard uncertainty in customer demand. In period *t*, we calculate the expected starting inventory for period t+τ, denoted by $E\left[I_{t+\tau }\right]$, based on the current inventory *i*_*t*_, previously determined replenishment order quantities $r_{t-\tau ,t},\ldots ,r_{t-1,t+\tau -1}$, expected demand in the meantime $\mu_{t},\ldots ,\mu_{t+\tau -1}$, the average shelf life, and the average amount of supply shortage. In the event of a fixed relative supply shortage, the retailer could simply add this percentage to the replenishment order quantity to ensure the intended amount is delivered. The order quantity under this deterministic approach is then given by the difference between expected demand and the expected starting inventory for the period under consideration, divided by the average relative amount of units supplied (note that we consider only positive replenishment order quantities):


$$r_{t,t+\tau }^{*}=\max\left(\frac{E\left[D_{t+\tau }\right]-E\left[I_{t+\tau }\right]}{1-\bar{\theta }},0\right).$$ (6)

Applying these point forecasts results in an average order quantity of 96.33, which is approximately 19% lower compared to the newsvendor approach. At the same time, the average inventory level of 18.93 is more than 90% lower. Consequently, the amount of spoilage is also significantly reduced. While this reduces operational costs for inventory holding and spoilage, the policy results in fulfilling 93.49% of total customer demand, which falls short of the retailer’s intended service level and evokes customer dissatisfaction. Thus, compared to the newsvendor approach, lost sales occur more frequently and account for a large share of total costs. However, total costs are slightly lower. By accounting for the inter-period dependencies in the inventory management framework through point forecasts on demand, shelf life, and supply shortages, the average per-period costs are reduced by 8.5% in total (see Table 2).

**Table 2:** Comparison of the lookahead policy to the myopic and deterministic approach.

setting	∅ order quantity	∅ inventory level	∅ amount of spoilage	% fulfilled demand	∅ costs per period
newsvendor	119.03	199.42	17.52	99.72%	38.84
point forecasts	96.33	18.93	0.99	93.49%	35.55
lookahead policy	103.05	59.16	3.53	98.47%	17.07

### 4.4 Evaluation of the lookahead policy

In our setting with multiple sources of uncertainty, using point forecasts reduces average per-period costs for perishable SKUs compared to the more myopic newsvendor model, which only addresses demand stochasticity. However, the approach presented in the previous section results in a higher level of unfulfilled demand than intended by the strategic service level of the e-grocery retailer. Therefore, we continue to seek a policy that effectively balances the trade-off between product availability and operational costs associated with excess inventory. For this purpose, we now apply the lookahead policy introduced in Section 3.5 to our simulated data set and evaluate the policy in detail. Since the outcome of any policy is highly dependent on the specific business case, this is followed by a discussion on the sensitivity of our results with respect to the underlying parameter values, thereby generalising to other inventory management settings.

In practice, a retailer must determine order quantities for all SKUs in the assortment daily within a limited time frame, which limits the computing power and time available for individual SKUs. To tackle this, we parameterise the policy based on a set of initial experiments, addressing the trade-off between computation time and stability of the simulation results. We use *N* = 1000 sample paths (simulation runs) for the stochastic lookahead while considering ν=3 additional periods with a weighting factor ρ=0.9. In each period, the retailer determines the replenishment order quantity according to the lookahead policy and the (known) probability distributions for each source of uncertainty. At the end of a period, we again use realised inventory holding, spoilage, and lost sales to calculate average per-period costs for the evaluation period.

Table 2 summarises the results of our analysis and compares them to those obtained under the newsvendor model and the deterministic approach. We find an average order quantity of 103.05, which is 7.0% higher than when relying solely on expected values. Meanwhile, the average inventory level is more than three times higher, although it remains only about 30% of the level observed under the newsvendor model. On average, 98.47% of customer demand is fulfilled, aligning with the retailer’s intended service level. The average per-period costs, when accounting for uncertainty in all three sources, are reduced by 52.0% compared to using expected values and even by 56.1% compared to the newsvendor model. This demonstrates that incorporating probabilistic information yields more accurate replenishment order quantities than both the myopic and deterministic approaches, successfully balancing customer satisfaction and operational costs associated with excess inventory.

In practice, retailers must accurately estimate the underlying probability distributions from often multidimensional historical data or features before they are able to make replenishment order decisions based on probabilistic information. These estimations require data collection, data preparation, and data analysis, incurring operational effort and costs for retailers, which must be considered. Addressing the research gap on targeted data utilisation identified by previous literature [19], we evaluate the benefit of applying distributional information in terms of EVIU, i.e., cost reductions achieved through precise distributional information, for each of the different sources of uncertainty (while limiting information on the other two sources to point forecasts). Table 3 presents information on the various settings compared to probabilistic information for all sources of uncertainty and also provides savings relative to the newsvendor model and the setting of point forecasts.

**Table 3:** Analysis of the expected value of including uncertainty (NV: newsvendor model; PF: point forecasts).

setting	∅ order quantity	∅ inventory level	∅ amount of spoilage	% fulfilled demand	∅ costs per period	cost change rel. to NV	cost change rel. to PF
distributional information for demand	103.86	60.72	3.37	98.5%	17.20	-55.7%	-51.6%
dist. info. for shelf life	96.92	20.11	1.06	94.0%	33.13	-14.7%	-6.8%
dist. info. for supply shortages	95.63	16.76	0.88	92.9%	38.08	-2.0%	+7.1%
dist. info. for each uncertainty source	103.05	59.16	3.53	98.5%	17.07	-56.1%	-52.0%

Incorporating distributional information solely for demand already results in a substantial reduction in total costs compared to point forecasts (−51.6%). To accommodate demand variability, the retailer increases replenishment order quantities and maintains a significantly higher safety stock. Consequently, the average inventory level and amount of spoilage increase more than threefold compared to the situation of point forecasts. However, because of the asymmetric cost structure, savings due to the enhanced service level and resulting customer satisfaction surpass additional operational expenditures associated with spoilage and inventory holding. Cost improvements are also observed when including the shelf life’s probability distribution only, albeit with a much smaller effect — a total cost reduction of 6.8% compared to using a policy based on point forecasts — due to the low probability of spoilage within the first two sales periods. In contrast, incorporating a probability distribution only for supply shortages results in a decrease in summary statistics on average order quantity, inventory level, and percentage of fulfilled demand. Specifically, the average order quantity of 95.63 (see Table 3) is lower than the corresponding value of 96.33 under the deterministic approach (see Table 2), where the retailer adds a fixed percentage to each order to account for expected supply shortages (see Eq (6)). Overall, the deterministic approach overcompensates for stochastic supply shortages in most periods, resulting in a 13% increase in the average inventory level. Consequently, the higher inventory level, due to less information on supply shortages, leads to increased holding and spoilage costs. However, the increased inventory level also has a positive effect, as indicated by the comparatively higher percentage of fulfilled demand in the deterministic case: it unintentionally increases customer satisfaction due to reduced lost sales, decreasing associated costs incurred by disregarding demand variation in the deterministic setting. Given the asymmetry of cost parameters, average total costs are lower for the less informed decision-maker. The results presented in this section are derived from simulation-based analyses and therefore depend on the underlying assumptions, such as demand following a negative binomial distribution. In practice, the magnitude of cost savings achievable through probabilistic information will vary with the validity of these assumptions as well as the accuracy of data collection and preparation processes. Accordingly, while our findings illustrate the potential benefits under controlled experimental conditions, the exact cost reductions realised in practice may differ depending on the retailer’s data environment and operational setting.

Table E1 in Supporting Information E presents additional results on scenarios where distributional information is applied for two sources of uncertainty while using expected values for the third source. Our findings indicate that the EVIU varies across the different model components, and that also the sequence of including distributional information matters. For instance, including the probability distribution for supply is beneficial only when the retailer also accounts for uncertainty in demand.

### 4.5 Sensitivity analysis

The simulation-based analysis above demonstrates how retailers can reduce total costs by using probability distributions instead of expected values for each source of uncertainty, assuming the underlying probability distributions are known. However, the results are likely to be highly dependent on the exact specification of the distributions of the random variables associated with demand, supply, and shelf life (and, of course, also on the cost structure). Therefore, we provide additional sensitivity analyses, considering variation in location and dispersion of the underlying probability distributions and parameters. For each of the three sources of uncertainty, we compare the results obtained using the deterministic approach (referred to as Scenario 1) with those obtained using full probabilistic information (referred to as Scenario 8) within the same simulation setting.

**Fig 4:**
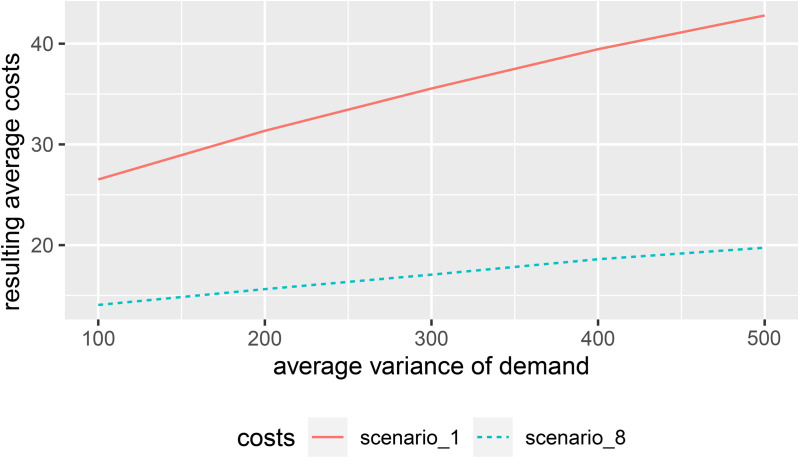
Resulting per-period costs depending on the variance of demand.

Given that our costs are asymmetric, with lost sales being more expensive than inventory and spoilage, we expect the benefit of incorporating the demand distribution to increase as its variance increases. We continue to allow for non-stationary demand but vary the parameter in the variance-generating Poisson distribution, $\lambda_{\kappa }\in \{100,200,300,400,500\}$, while keeping $\lambda_{\mu }=100$ constant. Fig 4 illustrates that average costs increase substantially when using expected values only (Scenario 1), while the per-period costs only slightly increase when incorporating full distributional information for all sources of uncertainty (Scenario 8). As expected, the importance of integrating information on the demand distribution increases with its variance.

For the sensitivity of the shelf life (for details see Supporting Information F in S1 Appendix), we find that including information on the shelf life distribution is most beneficial for distributions with high variance or a small mean (corresponding to a high risk of spoilage in early periods). The relevance of incorporating probabilistic information on potential supply shortages (for details see Supporting Information G in S1 Appendix) depends not only on the associated risk but also on the persistence of the corresponding process (i.e., whether shortages tend to occur in several consecutive periods).

Finally, we examine changes in the cost structure for lost sales, inventory holding, and spoilage. As previously mentioned, costs in e-grocery retailing are generally asymmetric due to the economic impact of lost sales. Therefore, the benefits of incorporating additional information on probability distributions are expected to diminish if cost parameters become more symmetric. We test this hypothesis by changing the relationship between cost parameters. While maintaining a constant relationship between inventory costs *v* = 0.1 and spoilage costs *h* = 1, we vary the costs for one unit of lost sales. In the first analysis, we assume that lost sales costs are equal to inventory costs, leading to *b*_1_ = 0.1. In addition, we consider *b*_2_ = 0.5, *b*_3_ = 1 (where the costs of lost sales and spoilage are identical), *b*_4_ = 2, *b*_5_ = 5, and *b*_6_ = 10.

**Fig 5:**
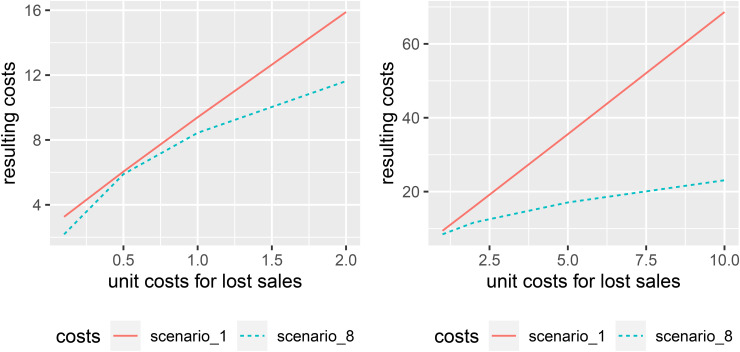
Resulting average per period costs depending on unit costs for lost sales.

Fig 5 illustrates average per-period costs for the deterministic approach (Scenario 1, red solid line) and full probabilistic information (Scenario 8, blue dashed line) across various unit costs for lost sales. When these costs fall between the unit costs for inventory holding and spoilage, the difference is negligible. However, incorporating probability distributions becomes more significant if the cost structure is asymmetric.

**Fig 6:**
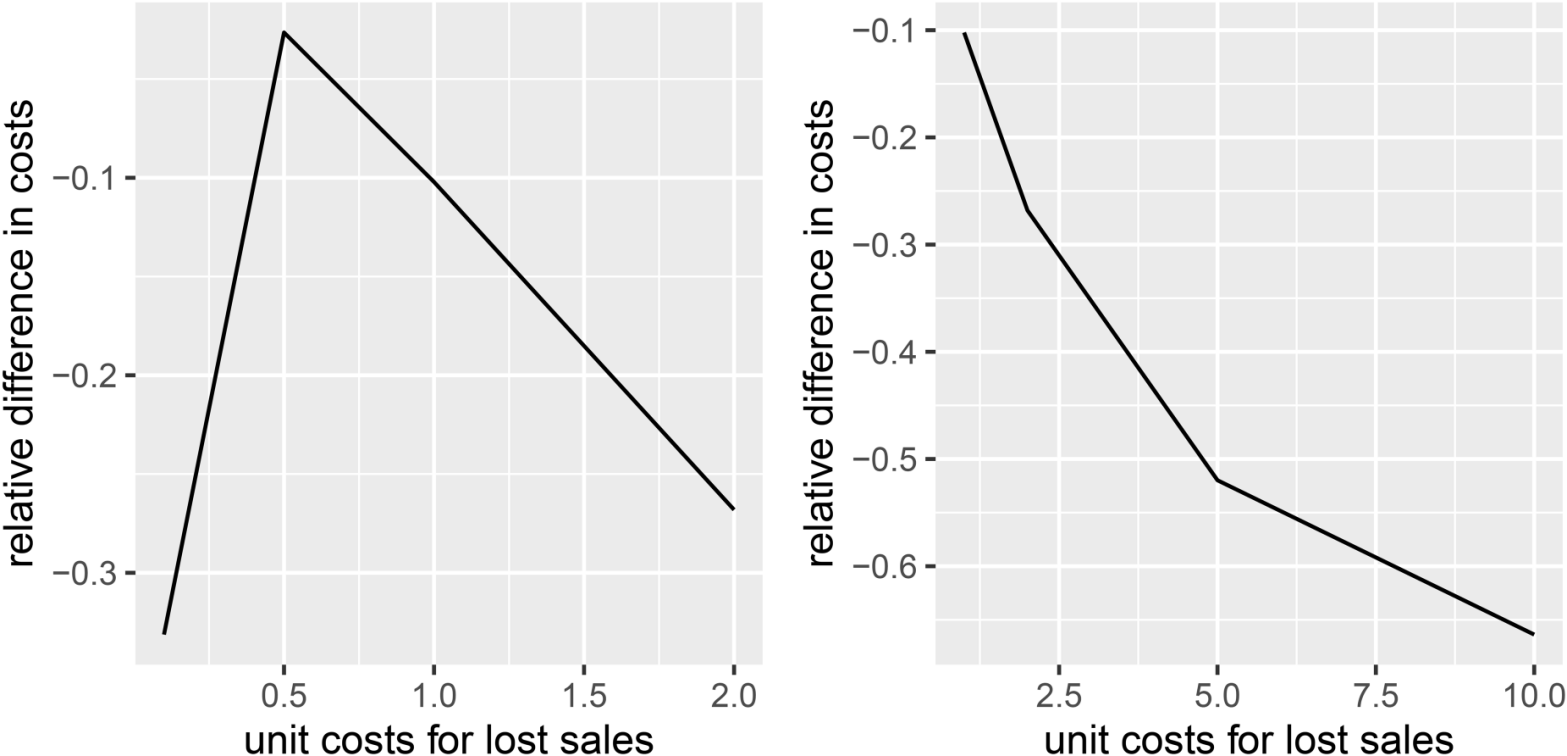
Relative difference in resulting average per period costs between Scenarios 1 and 8 depending on unit costs for lost sales.

This result is further supported by Fig 6, which shows the relative difference in average per-period costs. With *b* = 0.5, i.e., the cost per unit lost is half that of the cost per unit of spoilage, incorporating distributional information yields a saving of only 2.6%. In contrast, for the business case of e-grocery retailing with an asymmetric cost structure (and the corresponding high service-level targets), savings are significantly larger. As mentioned earlier, with costs per unit lost set at *b* = 5, including information on the distribution of demand, spoilage, and supply shortages reduces costs by more than 50%, and potential savings are even greater for *b* = 10.

## 5 Conclusion

In this paper, we explore how data-driven probabilistic information can help maintain a specified strategic service level while minimising operational costs in e-grocery retailing. We examine the inventory management problem of a major European e-grocer faced with non-stationary uncertainty in customer demand, stochastic shelf lives, random yield, and a (deterministic) lead time spanning several days. This necessitates an approach tailored to the requirements prevalent in e-grocery retailing practice, where retailers receive information updates regarding stochastic variables within each period. To determine replenishment order quantities, we propose a stochastic lookahead policy embedded within a data-driven sequential decision process. Our policy allows for integrating probabilistic forecasts for the underlying probability distributions into the optimisation process within a dynamic multi-period framework. Our goal is to understand the value of probabilistic information in our context, specifically to provide guidance for retailers on which sources of uncertainty warrant investments in comprehensive data collection and preparation. This addresses the question of targeted data utilisation raised by previous literature.

By evaluating a data set generated in accordance with data from retail practice, we analyse the value of explicitly exploiting probabilistic information rather than relying on point forecasts (expected values) in our replenishment decisions. Our results indicate that incorporating distributional information for all sources of uncertainty considered can lead to substantial cost reductions (with the amount of savings naturally depending on the specific situation). The importance of including distributional information tends to increase with higher asymmetry in cost parameters (i.e., very low or very high service-level targets), as commonly observed in e-grocery retailing. Regarding the different sources of uncertainty, results indicate that the benefit of integrating probability distributions instead of expected values is highest for customer demand. In contrast, the additional contribution of modelling shelf lives and supply shortages by probability distributions is marginal in the baseline case but highly dependent on the structure of the underlying probability distributions. Beyond explicitly accounting for all sources of uncertainty, a key advantage of our lookahead policy over simple parametric policies is its natural adaptability to changing environments (e.g., induced by dynamic market developments), structural shocks (e.g., the COVID-19 pandemic), and regime shifts due to strategic changes (e.g., an increased focus on sustainability). Furthermore, it can be easily adapted to the business cases of other companies. Specifically, our sensitivity analyses already provide a generalisation to other cases and present expected results in various settings.

Still, the results of our study are subject to limitations. First, unlike the newsvendor model, where a well-established analytical relationship links the cost ratio and the service level under the optimal solution, our multi-period decision model, solved using the lookahead policy, requires this relationship to be explored numerically. In our case, the retailer’s management formulates the relevant objectives for the e-grocery unit through a strategic service level target. Accordingly, in our numerical experiments, we assume a specific relationship between the lost-sales cost parameter and the other per-unit costs, based on insights from a set of initial experiments. Future research could further elaborate on the exact relationship within our modelling environment. Moreover, we simplified by disregarding more complex demand patterns such as seasonality and day-of-week effects. To more accurately capture the operational setting of grocery retailing, future work could relax the assumption of independent demand across periods. Our model, which can naturally accommodate structural changes in the information model, could then incorporate information derived from probabilistic demand forecasts [12].

Specifically, to address the issue of a case-specific quality of an estimation method in the information model [32], statistics suggest considering the loss function resulting from the decision problem as a benchmark. Therefore, to choose an appropriate method, the lost-sales cost parameter must be known, implying that optimising the decision model and selecting the estimation approach should be carried out simultaneously. This issue is also addressed in the outlook of [24], who suggest integrating parameter estimation into the optimisation of replenishment order quantities and is directly related to recent discussions on ‘predict-and-optimize’ (see, e.g., [49,71]; and [72] for an overview of research on the integration of inventory control and demand forecast). Furthermore, probabilistic forecasts based on contextual data, such as weather conditions, may enhance the power to explain underlying variation in the stochastic variables supply and spoilage. Given the flexibility of the policy proposed in this work, such an extension would also allow the integration of more complex patterns, such as trends and seasonality, without changing the properties of our policy.

## Supporting information

S1 Appendix Business case and further results. (PDF)
