# Supplementary material for: Managing inventories for perishable e-groceries: The value of probabilistic information
Source: PLoS One. 2026 Apr 9;21(4):e0343935. doi: 10.1371/journal.pone.0343935 (PMC13065039; doi:10.1371/journal.pone.0343935)
Supplement: S1 Appendix — (PDF) [file pone.0343935.s001.pdf]

S1 Appendix: Business case and further results

A Description of the business case

This paper is based on the business case of a major European e-grocery retailer ([12] and [8]). The retailer's assortment includes frozen products, those requiring refrigeration, as well as fruits and vegetables. A two-step logistics process is applied: initially, central distribution warehouses supply so-called fulfilment centres, from which purchases are then delivered directly to customers. In this paper, we take the perspective of a single fulfilment centre.

The data set provided by the e-grocery retailer encompasses demand periods of six different fulfilment centres for the period from January 2019 to December 2019, i.e. prior to the beginning of the COVID-19 pandemic. Each observation corresponds to a single demand period, representing a single day of delivery. For illustration, Fig A1 depicts the daily demand for the SKU *mushrooms* in 2019 at one selected fulfilment centre. We observe recurring peaks on Mondays, with no significant trends or seasonal patterns.

Fig A1. Realised demand for the SKU mushrooms in 2019.

In terms of forecasting demand based on features, the additional operational processes of picking and delivery extend the lead time compared to traditional brick-and-mortar retailing. Fig A2 demonstrates the impact of lead time by showing the mean average percentage forecast error as a function of the lead time, using linear regression for all SKUs within the categories fruits and vegetables for the demand period from January 2019 to December 2019. The forecasting accuracy, measured by the mean average percentage error, strongly decreases with increased lead time, as less demand information is available for longer lead times.

Fig A2. Mean average percentage error (mape) as a function of the lead time of the e-grocery retailer for all SKUs within the categories fruits and vegetables in the demand period January 2019 to December 2019.

To derive the stochastic distribution of the shelf life for the SKU in our simulation-based analysis, we consider the number of units spoiled at the end of a certain period, for which we calculate the supply date under the assumption of the FIFO principle. This approach enables us to derive the relative frequencies of shelf lives

within the data set. Fig A3 illustrates the estimated CDF of the shelf life again for the SKU *mushrooms*. We find minimal variation between months, indicating low seasonality in the shelf life of this SKU. Approximately 30% of the units have a shelf life of more than two days, while about half of the units spoil after just one day.

Fig A3. Estimated CDF for the shelf life of the SKU mushrooms for July-December, aggregated over all warehouses.

Furthermore, the data set includes information on the quantity ordered, the quantity delivered by the central distribution warehouse, and the number of units spoiled during a specific demand period. This enables us to derive transition probabilities for supply states based on the data in the business case.

B Numerical example on the dynamics of the inventory system

We consider an exemplary demand period t. We assume that the SKU under consideration has a shelf life of six periods. Initially, 50 units are held in inventory, with 10 units delivered by the supplier in period $t-2$ and 40 units in period $t-1$. This results in the inventory vector $\tilde{i}_t = (0, 40, 10, 0, 0, 0)$. We consider a replenishment order quantity $r_{t-\tau,t} = 60$ and assume a relative shortage of 20% for period t. This leads to a delivered quantity of $q_t = 48$ and the adjusted inventory vector $\tilde{i}'_t = (48, 40, 10, 0, 0, 0)$. We assume demand is $d_t = 46$. According to the FIFO principle, we primarily sell units from earlier periods, i.e. $t-1$ and $t-2$. This results in the inventory vector $\tilde{i}''_t = (48, 4, 0, 0, 0, 0)$. Finally, we assume that 2 out of 4 units from period $t-1$ deteriorate, while 12 out of 48 units delivered in period t deteriorate. This results in $z_t = 14$ and the final inventory vector $\tilde{i}'''_t = (36, 2, 0, 0, 0, 0)$ to be transferred to $\tilde{i}_{t+1} = (0, 36, 2, 0, 0, 0)$. The transitions are summarised in Table C1.

C State distribution of supply shortage

Let δ_t represent the proportion of the ordered quantity r_t that is actually supplied, so that $1-\delta_t$ indicates the relative supply shortage. The sequence of supply states

Table C1. Inventory vectors according to the example used for the illustration of the dynamics given in Supporting Information B.

action		$q_t = 48$	$d_t = 36$	$z_t = 14$
resulting inventory vector	$\tilde{i}_t$	$\tilde{i}'_t$	$\tilde{i}''_t$	$\tilde{i}'''_t$
entry				
$t, 0$	0	48	48	36
$t, 1$	40	40	4	2
$t, 2$	10	10	0	0
$t, 3$	0	0	0	0
$t, 4$	0	0	0	0
$t, 5$	0	0	0	0

$G_1, \ldots, G_T$ is governed by a homogeneous Markov chain with transition probabilities $\theta_{i,j} = \Pr(G_t = j | G_{t-1} = i)$, $i, j \in \{0, 1, 2\}$, $t \geq 2$. The state distribution for the first period $t = 1$ is assumed to follow the Markov chain's stationary distribution, $\boldsymbol{\theta}^* = (\Pr(G_t = 1), \Pr(G_t = 2), \Pr(G_t = 3))$. The proportion of units supplied δ_t is then determined as follows:

$$\delta_t = \begin{cases} 1 & \text{if } G_t = 1 \\ 0 & \text{if } G_t = 2 \\ \text{Beta}(\alpha, \beta) & \text{if } G_t = 3. \end{cases} \tag{7}$$

In the case of partial delivery, the beta distribution assumed for the proportion of units delivered implies a mean supply rate of $\alpha/(\alpha + \beta)$, where the sum $\alpha + \beta$ serves as a precision parameter. Across all three states, the proportion of units supplied follows a beta distribution, with additional point masses on zero and one, and a stationary mean of $\theta_1^* + \theta_3^* \cdot \alpha/(\alpha + \beta)$.

D Calculation of conditional probabilities for spoilage

The conditional probability p_j that a given unit deteriorates after j periods is defined as follows:

$$p_j = \begin{cases} f^{sl}(j) & j = 0; \\ \frac{f^{sl}(j)}{1 - F^{sl}(j-1)} & j > 0, \end{cases} \tag{8}$$

where f^{sl} is the probability function of shelf life and F^{sl} the corresponding CDF. The inventory is represented by a vector $\tilde{i}_{t,j}$, as introduced in Supporting Information B, to keep track of the different delivery periods of units in stock. Given the probability p_j, the number of units from a set of $\tilde{i}_{t,j}$ units, all with the same supply date and a shelf life of j, that deteriorate at a given day can be modelled by a binomial distribution with parameters $\tilde{i}_{t,j}$ and p_j. Therefore, the total number of deteriorated units at the end of period t, $Z(i_t)$, results from the joint distribution of these J binomial distributions for the elements of the inventory vector $\tilde{i}_{t,j}$, each with its own parameters. All remaining units are transferred to the next period.

Table D1. Conditional probability of spoilage p_j at the end of a given demand period j in the simulated data set.

demand period j	1	2	3	4	5	6
spoilage probability p_j	0.050	0.105	0.176	0.500	0.571	1.000

E Additional results on the effect of probabilistic information

Table E1. Statistics on the average order quantity, inventory level, amount of spoilage, fulfilled demand, and per-period costs for all scenarios.

	distributional information on			average	average	average	average	average
	demand	shelf life	supply	order quantity	inventory level	amount of spoilage	fulfilled demand	per period costs
Scenario 1				96.33	18.93	0.99	93.49%	35.55
Scenario 2			x	95.63	16.76	0.88	92.91%	38.08
Scenario 3		x		96.92	20.11	1.06	94.00%	33.13
Scenario 4		x	x	96.27	17.97	0.94	93.48%	35.44
Scenario 5	x			103.86	60.72	3.37	98.46%	17.20
Scenario 6	x		x	104.09	62.86	3.51	98.55%	17.07
Scenario 7	x	x		103.79	59.84	3.33	98.44%	17.16
Scenario 8	x	x	x	103.86	60.47	3.36	98.47%	17.07

F Sensitivity analysis on shelf life

The sensitivity of the results with respect to the shelf-life distribution is analysed in two different ways. First, we examine two settings (f_1^{sl} and f_2^{sl}) with the same mean shelf life (three periods) but different levels of variability. Second, we analyse two shelf-life distributions (f_3^{sl} and f_4^{sl}) that share a relatively small variance but have different mean shelf lives. The distributions are detailed in Table F1. Here, f_1^{sl} corresponds to an SKU with little variation in shelf life, where 70% of the units deteriorate no more than one

day after the expected shelf life, and each unit is saleable for 2–5 periods. In contrast, f_2^{sl} represents a heavy-tailed distribution where both short shelf lives (one period) and longer ones (six periods) are pretty likely. Distribution f_3^{sl} corresponds to a situation where 80% of the units spoil within the first two demand periods, with a mean shelf life of two periods, whereas f_4^{sl} has an average shelf life of five periods.

Table F1. Distributions of shelf life in the sensitivity analysis.

j	1	2	3	4	5	6
$f^{sl}(j)$	0.05	0.10	0.15	0.35	0.20	0.15
$f_1^{sl}(j)$	0	0.1	0.25	0.7	0.05	0
$f_2^{sl}(j)$	0.2	0.05	0.05	0.25	0.15	0.3
$f_3^{sl}(j)$	0.4	0.4	0.075	0.075	0.025	0.025
$f_4^{sl}(j)$	0.025	0.025	0.075	0.075	0.4	0.4

Table F2 provides an overview of the resulting average per-period costs under Scenario 1 (using expected values only), Scenario 3 (using distributional information for shelf life only), and Scenario 8 (using full distributional information). In the baseline setting, according to the data set introduced in Section 4.1, the distribution is nearly symmetric around the mean shelf life of three periods, with a small risk of spoilage within the first two periods. In this setting, incorporating full distributional information can achieve cost reductions of around 52%, whereas the reduction in Scenario 3 is limited to 6.8%. If the risk of very early spoilage is low, as indicated by a small variance (f_1^{sl}) or a high mean (f_4^{sl}), similar cost reductions are achieved. In contrast, incorporating distributional information for shelf life only (Scenario 3) is more beneficial for distributions with a high variance (f_2^{sl}) or a small mean (f_3^{sl}), which corresponds to a high risk of spoilage in early periods. At the same time, due to increased total costs, reductions achieved when incorporating probability distributions for all sources of uncertainty (Scenario 8) are smaller than under the baseline distribution.

Table F2. Comparison of resulting average per-period costs for Scenarios 1, 3, and 8 depending on the distribution of shelf life. Relative savings compared to the deterministic approach (Scenario 1) in brackets.

set	distribution of shelf life	deterministic approach	probabilistic information on shelf life	full probabilistic information
f^{sl}	baseline	35.55	33.13 (−6.8%)	17.07 (−52.0%)
f_1^{sl}	small variance	33.88	33.83 (−0.0%)	14.75 (−56.4%)
f_2^{sl}	high variance	40.01	34.05 (−14.9%)	22.38 (−44.1%)
f_3^{sl}	small mean	44.52	37.28 (−16.3%)	27.28 (−38.7%)
f_4^{sl}	high mean	34.81	33.28 (−4.4%)	15.63 (−55.1%)

G Sensitivity analysis on supply shortages

For supply shortages, we consider four different transition probability matrices regarding the change of supply states while holding the parameters of the beta distribution in case of a partial supply shortage constant. The first matrix corresponds to a situation where the retailer encounters complete shortage slightly more frequently than in the baseline scenario, with rare transitions to partial or full shortage:

$$\Theta_1 = \begin{pmatrix} 0.95 & 0.01 & 0.04 \\ 0.3 & 0.2 & 0.5 \\ 0.3 & 0.5 & 0.2 \end{pmatrix}, \quad \theta^* = (0.857, 0.062, 0.081)^t.$$

In the second setting, with

$$\Theta_2 = \begin{pmatrix} 0.8 & 0.199 & 0.001 \\ 0.199 & 0.8 & 0.001 \\ 0.495 & 0.495 & 0.001 \end{pmatrix}, \quad \theta^* = (0.4995, 0.4995, 0.001)^t,$$

Partial supply in the next period occurs with a probability of 0.001, regardless of the current state. The other two states, full supply and full shortage, occur equally often. In the last two settings, the stationary probabilities are identical across all three states. The difference between these two settings lies in the state persistence, with Θ_3 corresponding to higher and Θ_4 to lower persistence:

$$\Theta_3 = \begin{pmatrix} 0.9 & 0.05 & 0.05 \\ 0.05 & 0.9 & 0.05 \\ 0.05 & 0.05 & 0.9 \end{pmatrix}, \quad \pi^* = (1/3, 1/3, 1/3)^t,$$

$$\Theta_4 = \begin{pmatrix} 1/3 & 1/3 & 1/3 \\ 1/3 & 1/3 & 1/3 \\ 1/3 & 1/3 & 1/3 \end{pmatrix}, \quad \pi^* = (1/3, 1/3, 1/3)^t.$$

The results presented in Table G1 show significant variation in relative cost savings when comparing the resulting average per-period costs under the deterministic approach (Scenario 1) to those obtained under full probabilistic information for different

transition probability matrices on supply states (Scenario 8). Due to the increased risk of (partial) supply shortages, average total costs are higher in all cases considered here compared to the baseline matrix. This also leads to a decreased potential for cost reductions when incorporating probabilistic information for all sources of uncertainty. However, while the low risk of supply shortages in the baseline case even increases total costs in Scenario 2 (probabilistic information on supply only), we find cost reductions for all cases in this analysis. Since lost sales are more expensive than inventory holding and spoilage, a model that incorporates knowledge of the TPM determines replenishment order quantities such that a larger safety stock is maintained. Therefore, substantial cost savings can be achieved in Setting Θ_1. A similar result is obtained when considering Θ_4, where the probability of each of the three supply states is 1/3, independent of the previous state. At the same time, savings in Settings Θ_2 and Θ_3 are much smaller. Due to the persistence of the same supply state, the retailer is rarely able to respond to supply shortages by increasing the replenishment order quantity for the following period, as there remains a high probability of continued shortages.

Table G1. Comparison of resulting average per-period costs for Scenarios 1, 2, and 8 depending on the TPM of supply states. Relative savings compared to the deterministic approach (Scenario 1) in brackets.

set	deterministic approach	probabilistic information on supply shortages	full probabilistic information
baseline	35.55	38.08 (+7.1%)	17.07 (−52.0%)
Θ_1	56.97	42.96 (−24.6%)	42.96 (−24.6%)
Θ_2	195.15	188.48 (−3.4%)	182.81 (−6.3%)
Θ_3	164.28	156.82 (−4.5%)	156.62 (−4.7%)
Θ_4	116.05	72.05 (−37.9%)	70.67 (−39.1%)
